# Swedish Pharmacy Students’ Expectations and Perceptions of Their Education and Future Pharmacy Profession

**DOI:** 10.3390/pharmacy7040139

**Published:** 2019-09-25

**Authors:** Maria Gustafsson, Sofia Mattsson

**Affiliations:** Department of Pharmacology and Clinical Neuroscience, Umeå University, SE-901 87 Umeå, Sweden

**Keywords:** pharmacy, web-based education, students, perceptions, expectations, profession

## Abstract

Distance education is becoming more and more common, and today distance education is well established within academic settings. The aim was to investigate first-year pharmacy students’ expectations and perceptions of web-based pharmacy programs and of their future profession. Furthermore, student characteristics were compared over time. A questionnaire was distributed to all first-year students admitted to the pharmacy programs at Umeå University in 2017. The students were asked questions about their background, motives for choosing pharmacy education, and their expectations and perceptions of their education and profession. Factors of most importance when choosing the education were: the education is interesting, leads to an interesting job, and is web-based. The students’ expectations of the education were high, and they want to learn as much as possible and be well prepared for their future profession. Regarding the students’ perception of their future profession, three themes were identified: to help other people, professional development, and employment related issues. Student characteristics have changed over the years, suggesting that the web-based pharmacy education and the flexibility it entails attracts other groups of students today compared with when the programs started.

## 1. Introduction

Delivering education using a web-based format is becoming more and more common, and today distance education is well established within academic settings in both Sweden and elsewhere. The number of students enrolled in distance education in Sweden was 111,900 in 2017/18, and this accounted for 28% of all university students [1]. The number includes both those studying only distance education and those combining distance and campus-based education. The corresponding number of students registered only in distance education was 76,600 (19%). Previous studies have found that web-based education attracts certain type of students, e.g., students who are generally older, have dependent children, and are more likely to be female compared to students studying on campus [1,2,3].

Web-based education has been shown to offer flexibility, facilitating the combination of university studies with life and job commitments [4,5,6]. Indeed, flexibility is often stated as one major reason for choosing a web-based format [2]. Web-based education might, among other initiatives, also help address the uneven distribution of health-care professionals by offering flexible education opportunities [7]. An uneven distribution of health-care professionals e.g., doctors, nurses, and pharmacists, leads to a shortage of these professions in, for example, rural areas. 

There are two different professional degrees within pharmacy in Sweden—prescriptionists and pharmacists, as seen in Table 1. Prescriptionists hold a three-year degree (bachelor) in pharmacy, and pharmacists hold a five-year degree (master) in pharmacy. In 2003, the Bachelor of Science of Pharmacy program was introduced at Umeå University, and this was the first web-based pharmacy program in Sweden. The program was introduced as a response to the observed shortage of prescriptionists in Northern Sweden [7]. A web-based approach was chosen in order to attract eligible students in rural areas as well as to achieve equity in terms of allowing different groups to commence higher education. This program was followed by the development of a Master of Pharmaceutical Science program in 2010 and a Master of Science in Pharmacy program in 2012. The Master of Pharmaceutical Science program is intended for people who already hold a bachelor’s degree in pharmacy and who are wanting to pursue a master’s degree in pharmacy. Students admitted to the Master of Science in Pharmacy program are guaranteed to be able to continue to the master’s level if the prerequisites are fulfilled, whereas, students with a bachelor’s degree in pharmacy apply separately to the master’s level through the Master of Pharmaceutical Science program.

Previous investigations of students graduating from the Bachelor of Science of Pharmacy program at Umeå University have shown that they were very satisfied with their education, as well as with their jobs as practicing prescriptionists [8,9]. Past studies regarding students’ perceptions of their curriculum and profession have dealt with, for example, factors influencing the choice of pharmacy as an education and factors affecting the perception of the professional role of pharmacists [10,11]. Students have reported many different reasons for choosing pharmacy education such as interests in natural science, a desire to help people improve their health, the influence of family members and friends, a desire to get a job with high salary, and the possibility of making a successful career [12,13,14]. Furthermore, factors such as gender, prior work experience in pharmacy-related areas, and acquired background knowledge influenced the students’ perceptions of their professional role [11]. However, little is known of Swedish pharmacy students’ expectations and perceptions concerning their education and future profession upon enrolment. Furthermore, because distance education is becoming more common, the characteristics of enrolled students are likely to change.

The aim of this study was to investigate first-year pharmacy students’ expectations and perceptions of their pharmacy program and future pharmacy profession. The students were enrolled in web-based pharmacy programs. Furthermore, the aim was to compare the characteristics of students enrolled in web-based pharmacy education over time, i.e., to compare the first cohort of 2003 with students admitted in 2017.

## 2. Materials and Methods 

### 2.1. Setting

A study questionnaire was developed based on the results of focus group interviews with students admitted to the Bachelor and Master of Science in Pharmacy program, as well as students admitted to the Master of Pharmaceutical Science program. The purpose of the questionnaire was to explore student’s expectations and perceptions of the web-based pharmacy program at Umeå University and of their future pharmacy profession. The questionnaires were distributed to all first-year students in these programs. The pharmacy programs at Umeå University are delivered in a web-based format using a virtual learning platform. The online material consists of recorded and streamed lectures, assignments, quizzes, animations, and simulations. Approximately 2–4 mandatory meetings (generally 3–5 days each) are held on campus each semester. During these meetings the students mainly train communication and laboratory skills. None of the pharmacy programs at Umeå University are offered as campus-based programs.

### 2.2. Focus Group Interviews 

Convenience sampling was used to identify first-year students on the Bachelor and Master of Pharmacy programs and the Master of Pharmaceutical Science program. An invitation to participate in focus group interviews was e-mailed to all students registered in the pharmacy programs in order to aid the development of a study questionnaire. Based on a previously used questionnaire [15] and discussions within the research team, an interview schedule with a list of topics was developed. The schedule focused on the students’ perceptions and expectations within the following four main areas: (1) why the students have chosen a pharmacy program; (2) why the students have chosen a web-based education; (3) why the students have chosen Umeå University (besides the web-based factor); and (4) the students’ opinions regarding the structure of the web-based education. During the interviews it was also possible for the students to express their own concerns. Seven students from the Bachelor and Master of Pharmacy programs and two students from the Master of Pharmaceutical Science program agreed to participate. Semi-structured interviews with these two groups of students were carried out at two different occasions in December 2017 in conjunction with a course meeting on campus. 

### 2.3. Study Questionnaire 

The study questionnaire was developed using information from the focus group interviews as well as a previously used questionnaire [15]. The questionnaires for the Bachelor and Master of Science in Pharmacy students and Master of Pharmaceutical Science students consisted of 26 and 24 questions, respectively. Both questionnaires were divided into the following five sections that included both open-ended and closed-ended questions: (1) student characteristics and demographics; (2) work history prior to beginning studies; (3) choice of education and expectations; (4) future professional life; and (5) an open-ended question where the students were asked to provide further comments. Section 3 included the two open-ended questions “How do you perceive the setup of the web-based education?” and “What are your expectations concerning your education?” Students admitted to the Master of Pharmaceutical Science program were also asked the following question: “Why did you chose to continue your education?” Section 4 included the single open-ended question, “What are your expectations concerning the future profession?” In Section 5, the students were asked to grade each factor for the question “Which factors have been important to you when choosing the education?” This was done using a Likert scale. The results are presented as mean values. 

The survey was distributed in February 2018 to first-year students admitted to the Bachelor and Master in Pharmacy programs during an on-campus meeting at the university. Of 48 students, 32 (67%) answered the questionnaire. The 48 students were registered in the course taken by first-year students at the time of the survey (29 were admitted to the Bachelor of Science in Pharmacy program and 19 to the Master of Science in Pharmacy program). Likewise, the survey was distributed in March 2018 to first-year students admitted to the Master of Pharmaceutical Science program during an on-campus meeting at the university. Of 18 students registered in the course given at that time, 15 students (83%) answered the questionnaire. 

### 2.4. Comparisons between the 2003 and 2017 Cohorts 

The results of the questionnaires were compared with a previous questionnaire distributed in 2003. Students admitted to the Bachelor of Science in Pharmacy program in 2003 were asked to participate in a survey during their first semester. Of 109 students enrolled in the program, 108 students (99%) competed the questionnaire. The survey contained questions about the students’ backgrounds, living conditions, choice and expectations of the education, and expectations of future work settings. 

### 2.5. Statistics 

Descriptive statistics were used to summarize the quantitative data. Student’s *t*-tests were used to calculate *p*-values when comparing mean values between the different groups. Chi-squared tests were conducted to calculate *p*-values when comparing proportions of students in the 2003 and the 2017 cohorts. A *p*-value less than 0.05 was considered statistically significant. To the question, “Which factors have been important for you when choosing the education”, the students ranked the importance of the factor according to a 6-point Likert scale (0 = irrelevant, 1 = not so important, 2 = important, 3 = quite important, 4 = very important, and 5 = crucial). These factors were analyzed in a logistic regression model. In the model, the variables were dichotomized into “not important” (responses 0–2) and “important” (responses 3–5). The model used each factor as the dependent variable and included age and sex as independent variables. Open-ended responses regarding the questions “What are your expectations concerning your education” and “What are your expectations concerning the future profession” were analyzed using a modified thematic analysis, which involved an open coding technique. The open-ended questions “How do you perceive the setup of the web-based education” and “Why did you choose to continue your education” were not thematically analyzed because of similarities in answers and because the answers tended to be very brief. Responses were collected and analyzed using SPSS version 23.

### 2.6. Ethics 

No ethical committee approval was sought prior to beginning this research because it is not obligatory under Swedish law for this type of study. All data were anonymized before analysis. The participants in this study were informed about de-identification of the material and about the aim of the study, and by handing in the questionnaire they consented to the data being used for research purposes. 

## 3. Results

### 3.1. Student Characteristics in 2017

The total number of students who completed the questionnaires in 2017 was 47, giving an overall response rate of 71% (47/66), and all responses were included in the analysis of the results. The characteristics of the respondents are shown in Table 2. For comparison, gender and age were also obtained from the university admissions data. The majority of respondents were female (72%), and most students were enrolled in the bachelor program. The mean age of all students was 28.7 years. The median age varied between 23 and 34 in the different groups. The youngest were those admitted to the Bachelor and Master of Science in Pharmacy program. The difference in mean age between these two groups was not statistically significant (*p* = 0.21). The oldest were enrolled in the Master of Pharmaceutical Science program (*p* = 0.025 versus Bachelor of Science in Pharmacy and *p* < 0.001 versus Master of Science in Pharmacy when comparing mean values). The majority of the Bachelor of Science in Pharmacy students participating in the survey were born in Sweden, in contrast to the other programs. Furthermore, most students had either been studying at upper secondary school or been working prior to applying to their respective programs. In addition, a majority of respondents combined their studies with work commitments, and the extent of this varied between a few hours per week up to full-time (40 h per week). 

### 3.2. Comparison of Student Characteristics in 2003 and 2017

When comparing the student characteristics in 2003 and 2017, the students were younger in 2017 (*p* = 0.009) and to a lesser extent female (*p* = 0.027), as seen in Table 2. Furthermore, more respondents admitted to the bachelor program in 2003 had dependent children (*p* = 0.015) and had permanent employment at the time of admission (*p* = 0.028). In 2003, a majority of the students lived in Northern Sweden, whereas, in 2017 the majority lived in Southern Sweden (*p* = 0.001). Both in 2003 and 2017, a majority of the students had been working prior to applying to university (*p* = 0.408) and had chosen the education as their first choice (*p* = 0.078).

### 3.3. Education

**Figure 1:**
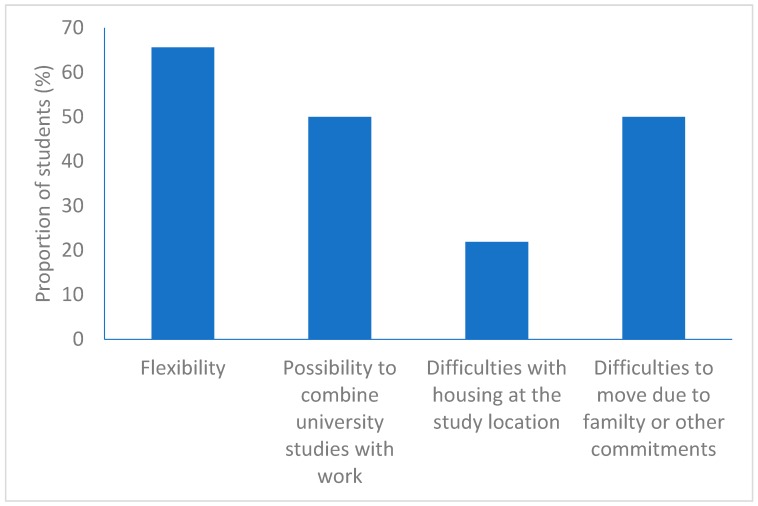
Reasons for choosing distance education. The students could choose several alternatives.

Regarding the reasons for choosing a web-based education, most students answered that it gave them flexibility, that it gave them the possibility to combine university studies with work, and that it allowed them to remain in their hometown due to family or other commitments (Figure 1). A majority of students admitted in 2017 and participating in the survey stated that they would not have applied to the program if it had not been offered as a web-based education (70%). In the 2003 cohort, this reason was not as common (44%) (*p* = 0.029).

Factors of importance to the students when choosing their education are presented in Table 3. The most important and second most important factor for respondents in all three programs was that the education is interesting and leads to an interesting job. That the program is web-based was the third most important factor for the Master of Science in Pharmacy students, while the students in the Bachelor of Science in Pharmacy program in 2017 rated “gives me personal development” and “gives me a university degree”, as more important factors. Students admitted to the bachelor program in 2003, rated “the possibility to obtain a job near my hometown” higher than the other students. The factors: “gives me personal development” and “a wide-ranging education” were rated as important for Master of Pharmaceutical Science students participating in the survey. This is in agreement with the results from the open-ended question that was answered by the students admitted to the Master of Pharmaceutical Science program: “Why did you choose to continue your education?” Almost all of the students answered that they wanted to broaden their competence and increase their opportunities for other workplaces. Comments were, for example, “I want to broaden my competence and take on new positions in the workplace such as working as a clinical pharmacist” and “to broaden my opportunities for work in government and industry”. Furthermore, the opportunity to work in their country of birth was also mentioned. No significant results were found in the regression analysis comparing those who ranked the different factors as “of no importance” or “of importance” regarding age and sex.

The analysis of the open-ended responses to the question, “How do you perceive the setup of the web-based education” showed that most students were satisfied with the setup. However, one student commented that this varied between the courses in the program, and another student commented that the setup was good, but it could be improved.

From the analysis of the open-ended responses to the question “What are your expectations concerning your education”, the following two broad themes were identified: (1) learning skills and (2) being prepared for the future profession. Regarding theme 1, students mentioned that they wanted to learn as much as possible. Among the comments were “to learn a lot and get a broad understanding of pharmacy” and “to have a good understanding of the body, diseases and treatments”. Regarding theme 2, students commented on their future job—“that I’ll learn a lot and feel competent when I’m looking for a job”, “be well prepared to become a clinical pharmacist”, and “get a new exciting job, perhaps become a PhD student”.

### 3.4. Future Profession

Students were also asked where they wanted to work after graduation, and most respondents admitted to the Bachelor of Science of Pharmacy program, in both 2003 and 2017, wanted to work at a community pharmacy. Also, a majority the respondents admitted to the Master of Pharmaceutical Science program had worked at a community pharmacy prior to admission. Among students admitted to the master’s programs, a greater diversity was observed, and the pharmaceutical industry, hospitals, and government were also considered to be of interest as future workplaces. Furthermore, most of the respondents in the 2017 cohort stated that they would be willing to move from their current place of residence after graduation.

Finally, all students were asked the open-ended question “What are your expectations concerning the future profession?” From the analysis of the open-ended responses to this question, the following three broad themes were identified: (1) to help other people, (2) professional development, and (3) employment-related issues. Within theme 1, the respondents explained that they wanted to help other people, which is exemplified by the comments “to meet other people and be able to help them” and “to help people feel as well as possible, to meet a lot of people and contribute to the workgroup and work”. Within theme 2, students mentioned that they, for example, expect “personal development and variation”. One student mentioned that they expect “an interesting job with many possibilities for development and career advancements”. Among employment-related issues (theme 3), the students mentioned, for example, “secure employment” and “a good working environment”.

## 4. Discussion

This study described first-year pharmacy students’ expectations and perceptions of a web-based pharmacy program and their future pharmacy profession. Furthermore, the study compared the characteristics of bachelor students admitted in 2003 with those admitted in 2017.

### 4.1. Student Characteristics

The majority of students studying pharmacy were female. This was especially evident among students admitted to the bachelor’s program and the two-year master’s program, whereas, the gender distribution was more equal among students admitted to the five-year master’s program. This is consistent with the pharmacy workforce in Sweden [16]. In 2017, 71% of the licensed pharmacists and 96% of the licensed prescriptionists in Sweden were female [16]. Also, a report from UK showed that there was a higher proportion of women than men in the pharmacist workforce [17]. According to Statistics Sweden, the proportions of women and men graduating with a pharmacy degree in 2017/2018 were 74% and 26%, respectively [18]. Corresponding numbers for the prescriptionist degree were 86% women and 14% men. In total in Sweden, 74% of the students admitted to the master’s program (pharmacist) and 79% of the students admitted to the bachelor’s program (prescriptionist) were women in 2017/2018. A study from a university in Canada showed that 74% of the pharmacy students were female [19], which was similar to other pharmacy schools in North America [20]. Overall, the trend over time is that the proportion of women studying to become pharmacists is increasing, whereas, the proportion of men studying to become a prescriptionist is increasing in Sweden [17]. This trend was confirmed when comparing the gender distribution between the cohorts of 2003 and 2017.

The median age of students admitted to the three-year bachelor’s and the five-year master’s program at Umeå University (Table 2) was slightly higher than the median age of pharmacy students in Sweden (22 and 21 years upon admission to a three-year bachelor’s and a five-year master’s program respectively) [1]. A recent study showed that the average age of students upon admission to a pharmacy program in Canada between 2002 and 2015 was 21.7 years [19]. The results in the present study are in agreement with previous studies reporting that students enrolled in web-based education are older than students enrolled in campus-based education [1,2,3,7]. The age structure was somewhat different between the groups admitted in 2017. Students admitted to the three-year bachelor’s and the five-year master’s program were younger than those commencing the two-year master’s program. Not surprisingly, students with a bachelor’s degree pursuing a master’s degree were older than the other students due to the fact that they had been studying and working for a number of years prior to pursuing a higher degree. The age distribution can also explain why the proportion of students having dependent children varied between the groups.

The students in 2017 were younger, and fewer students had dependent children compared to 2003. In a previous study analyzing the change in age at enrolment between 2003 and 2011, the average age at admission was 32 years, and it varied between 30 and 33 years during the period [7]. Apparently, the students admitted in 2017 were younger than before. Traditionally, distance education has been shown to attract older students [1,2,3,7]; however, as distance education is becoming more common it might become a more acceptable alternative to campus-based education also among younger students, and thus a decrease in age might not be so surprising. Looking at the application numbers for pharmacy programs in Sweden in 2017, the universities offering distance-based bachelor pharmacy programs had the highest application numbers [21] with approximately 2–3 applicants per available space. Apparently, the demand for a distance education alternative is high among the students.

In 2017, there were not as many students from Northern Sweden enrolled in the programs as compared to 2003. The reason for this is probably the organization of the program with local study groups as described previously [7]. In 2003, local study groups were located at different places in Northern Sweden. Approximately 1–2 times a week, these local study groups gathered students living in the same area for educational activities. As a complement to these local study groups, a distance group was also available, and the weekly meetings were conducted online instead. All students had access to the online learning material. Consequently, a majority of the students in the 2003 cohort lived in Northern Sweden because this was where the local study groups were located and people living in these regions were eligible to apply. This has been shifted towards southern Sweden because the local study groups are no longer offered by Umeå University. As the geographical distribution changes, this may affect the workforce and the allocation of prescriptionists/pharmacists in the country. Due to a longer distance to campus, the web-based format is probably more attractive to the students, and a smaller proportion of students in the 2017 cohort said they would have chosen the program if it had not been web-based compared with the 2003 cohort. Even though the possibility of obtaining a job near one’s hometown was ranked relatively high, the results showed that a majority (65%) of the bachelor students in the 2017 cohort were indeed willing to move after graduation in order to get a job. A previous study regarding the 2003 cohort found that the students to a greater extent wanted to work where they lived at the time of admission [15], suggesting that the mobility of the students is higher today. 

A majority of the students participating in the survey stated that they combined studies at the university with work. This is likely to be due to the web-based structure of the program facilitating studies to be combined with work and/or family commitments. Flexibility was also an important factor for choosing distance education. This could perhaps pose a problem for the educators because the students, due to other commitments, might not be able to fully focus on their studies and put in the time required to successfully pass the course. A previous study found that the overall throughput among bachelor pharmacy students at Umeå University was 74% (i.e., the proportion of admitted students who graduate) but that some students needed more time than the stipulated time frame before graduating, thus resulting in a considerable range in time to graduation [7]. The throughput is rather high considering the web-based format and comparable with many campus-based programs [22]. Web-based programs generally have a lower throughput compared to campus-based programs [23,24,25].

### 4.2. Education

The reasons for choosing a web-based education, i.e., flexibility, the possibility to combine university studies with work, and because of difficulties in moving to the place of the university due to family or other commitments in their hometown, are in line with previous research and reports [2,4,5,6]. The students appear to be satisfied with the setup of the web-based education, including the structure of the program, the pedagogical setup, and the online material. However, the comments were few and also brief and scanty, thus making it difficult in this study to obtain a thorough understanding of the students’ views on the setup in order to aid in educational development. There are also other sources of student feedback in the form of course and program evaluations, and these are useful when developing and improving the programs and the online material. 

When applying to university in Sweden, the students can rank their options according to their preferences. In this study, most of the students had chosen the pharmacy education at Umeå University as their first choice, indicating that this particular education (including the web-based format) and profession was their primary choice, which in turn might explain the student’s overall positive attitudes regarding their education and future profession. The analysis of the question regarding expectations for their education showed that the students’ expectations are high, and they want to learn as much as possible and be well prepared for their future profession.

Although the web-based format of the education appears to be appealing to the students, the factor of most importance to the students when choosing their education was not primarily the web-based format, but rather that the education is interesting and leads to an interesting job, and this was the case for all three programs. Similar results have been obtained previously [12,13,14]. The factors “gives me personal development” and “a wide-ranging education” were rated as important for students enrolled in the Master of Pharmaceutical Science program, and this is in line with the answers from the open-ended question where students expressed that they wanted to broaden their knowledge and skills as a reason for why they chose to continue their education. As a consequence, the students stated that they wanted to work at other workplaces after graduation, e.g., the pharmaceutical industry or hospitals, as opposed to community pharmacy where a majority of the students worked prior to applying to the Master of Pharmaceutical Science program. Therefore, it is important that the university is able to offer the possibility of further education in order to facilitate the students’ personal and professional development. A licensed prescriptionist is able to work in community pharmacy in Sweden, Finland, and Norway. Other countries do not have an equivalent degree, so in order to be able to work in other countries a master’s degree (pharmacist) is required. This was also stated as a reason for continuing one’s education to obtain a master’s degree. This interest might also be a reflection of the fact that a majority of the students obtaining a master’s degree are born outside of Sweden. Therefore, a master’s instead of a bachelor’s education might be considered as more appealing for some students. In contrast to a previous study, the students in the present study did not rank salary as especially important [26].

### 4.3. Future Profession

The major workplace for prescriptionists in Sweden is in community pharmacies, and a majority of the bachelor students, in both 2003 and 2017, wanted to work in community pharmacy after graduation. Among the master’s students, a greater diversity was observed regarding future workplaces. Regarding the students’ future profession, one factor of highest importance when choosing an education was that it would lead to an interesting job. The students indeed seem to have high expectations of their future profession; they are expecting a stimulating job with a high degree of responsibility, to contribute to health care, to help people, and to improve the use of pharmaceuticals. This is in agreement with previous studies [14,15,16]. In a previous study, the pharmacy graduates at Umeå University were very satisfied with their work experience [9], suggesting that these high expectations among the students may be fulfilled. However, other studies have shown a lower job satisfaction among pharmacists [27,28,29,30]. Comparisons with other countries may be difficult due to different contexts but there is limited information about job satisfaction among prescriptionists and pharmacists in Sweden. Furthermore, because the pharmacy profession is constantly changing, it would be interesting to follow up on job satisfaction over time and to investigate whether the expectations the students had when starting their education were fulfilled or not once they started their professional career. Many of the students expressed that they wanted to work with clinical pharmacy, both as a reason for why they choose to continue their education and as an expectation for a future job. Clinical pharmacy is an area that has been established for many years in countries such as USA and UK [31], and it is currently expanding in Sweden. 

Knowing which factors that are important for the students in their choice of education may be helpful when dealing with issues regarding development of the curriculum and educational activities, as well as recruitment. Since a wide-ranging education is considered important to students, it is important to continue to develop the programs in that direction and thus to prepare the students for work opportunities in different fields of pharmacy and pharmaceutical science. Also, flexibility is considered important when choosing a web-based format, and it is desirable to continue to offer the programs in this flexible manner. Furthermore, this study found that many of the students were interested in and wanted to work within clinical pharmacy, an area that was not at all in focus when the education started in 2003. This needs to be taken into account when planning future courses. 

This study asked first-year students about their expectations and perceptions. As discussed by, for example, Keshishian and Benton, students’ perceptions of their education and future profession might change during the course of their studies [13]. Nonetheless, by gaining an understanding regarding the students’ expectations and perceptions, educators will be able to better prepare the students for their future professional lives and will be able to develop strategies to address students’ perceptions. In professional degree programs, close alignment between the education and the profession is important in order to prepare the students for their future job assignments and to ensure that students obtain an accurate perception of the chosen profession during their education. Understanding students’ expectations and perceptions will also enable educators to work more effectively with recruiting and retention strategies as well as to address issues around curriculum planning and the development of educational activities. 

### 4.4. Limitations

This study has its strengths and limitations. One strength is that the study enabled a comparison of student characteristics over time. One limitation is selection bias, and the students choosing to complete the questionnaire might have been different from those who did not participate. The response rate was relatively high, which was probably due to the fact that the questionnaires were handed out during an on-campus meeting. The question regarding in which area the students wanted to work after graduation was misinterpreted by some students. The question explicitly asked for one alternative, the alternative the students thought was most suitable for them. However, some students chose several alternatives, which might have affected the interpretation of the results. Furthermore, the study was limited to first year students in order to explore the students’ perceptions and expectations in the beginning of their education and also to be able to compare the data obtained in 2017 to the data of 2003. No comparisons were made between web-based and campus-based programs perhaps making it difficult to extrapolate the results to students in general. Such a comparative study would be interesting to perform, in order to more thoroughly investigate differences and similarities between students enrolled in web-based and campus-based pharmacy programs. 

The size of the cohorts of 2003 and 20017 differed due to a change in the number of admitted students. The cohort of students admitted to the bachelor program in 2017 was small compared to 2003, and for that reason it might be more representative to compare gender and age using university admissions data. In the entire cohort of bachelor students in 2017 (*N* = 29), the mean age was 27.8 years and 82.8% were female, suggesting that the students completing the survey were representative of the entire cohort when it comes to gender and age. Other comparisons were made based on the results from the survey because those data were not available in the university admissions system.

## 5. Conclusions

This study explored first-year pharmacy students’ expectations and perceptions of a web-based pharmacy program and of their future pharmacy profession. Furthermore, the study investigated the characteristics of students enrolled in web-based pharmacy education over time. Factors of most importance when choosing the education were that the education is interesting, leads to an interesting job and is web-based. An interesting education was ranked higher than the web-based format. The students’ expectations of the education were high, and they want to learn as much as possible and be well prepared for their future profession. Regarding the students’ perception of their future profession three themes were identified: to help other people, professional development and employment related issues. The students enrolled today are younger than in 2003, suggesting that distance education and the flexibility it entails, might be an attractive alternative to campus-based education also among younger students.

## Figures and Tables

**Table 1:** Overview of academic and professional degrees offered at Umeå University.

Program	Years	Academic Degree	Professional Degree
Bachelor of Science in Pharmacy	3	BSc Pharm	Prescriptionist
Master of Science in Pharmacy	5	MSc Pharm	Pharmacist
Master of Pharmaceutical Science	2	MSc Pharm	Pharmacist

**Table 2:** Characteristics of the students participating in the surveys. Students were admitted to the Master of Science in Pharmacy program, Bachelor of Science in Pharmacy program and Master of Pharmaceutical Science program at Umeå University in August 2017. For comparison, characteristics of students admitted to the Bachelor of Science in Pharmacy program in 2003 are shown.

	Master of Science in Pharmacy (*N* = 12)	Master of Pharmaceutical Science (*N* = 15)	Bachelor of Science in Pharmacy (*N* = 20)	Bachelor of Science in Pharmacy 2003 (*N* = 108)
Female *N* (%)				
Respondents in the survey	7 (58.3)	11 (73.3)	16 (80.0)	102 (94.4)
From university admissions data	12/19 (63.2)	15/18 (83.3)	24/29 (82.8)	103/109 (94.5)
Age				
Mean ± SD (range) respondents in the survey	23.9 ± 5.0 (19-32)	36.5 ± 8.0 (24-51)	25.6 ± 7.4 (19-47)	32 *
Mean ± SD (range) from university admissions data	24.7 ± 6.5 (19-42)	33.6 ± 7.1 (24-50)	27.8 ± 9.1 (19-51)	32.4 ± 8.0 (19-56)
Median from university admissions data	23	34	25	33
Region of living *N* (%)				
Northern Sweden	1/11 (9.1)	0 (0.0)	10 (50.0)	90 (83.3)
Southern Sweden	10/11 (90.9)	13 (87.7)	10 (50.0)	18 (16.7)
Other countries	0/11 (0.0)	2 (13.3)	0 (0.0)	0 (0.0)
Country of birth *N* (%)				
Sweden	3/11 (27.3)	3 (20.0)	16 (80.0)	N/A
Rest of the world	8/11 (72.7)	8 (80.0)	4 (20.0)	N/A
Dependent children (yes) *N* (%)	1 (8.3)	10 (66.7)	5 (25.0)	59 (54.6)
Occupation before applying to the program *N* (%)				
Working	4/11 (36.6)	12/15 (80.0)	13/19 (68.4)	63 (58.3)
Studying at university	0/11	0/15	1/19 (5.3)	N/A
Studying at upper secondary school	5/11 (45.5)	1/15 (6.7)	4/19 (21.0)	N/A
Parental leave	0/11	2/15 (13.3)	0/19	N/A
Other (sick leave, sabbatical)	2/11 (18.2)	0/15	1/19 (5.3)	N/A
Permanent employment at time of admission (yes) *N* (%)	4/7 (57.1)	10/14 (71.4)	6/18 (33.3)	44/63 (69.8)
Left the permanent employment completely (yes) *N* (%)	2/5 (40.0)	4/14 (28.6)	7/13 (53.8)	22/63 (34.9)
Reasons for leaving the employment *N* (%)				
Due to termination of the employment	1/2 (50.0)	N/A	1/12 (8.3)	8/63 (12.7)
Due of health and/or working environment issues	0/2 (0.0)	N/A	2/12 (16.7)	6/63 (9.5)
Chose not to continue	1/2 (50.0)	N/A	9/12 (75.0)	45/63 (71.4)
Previously studied at a university (yes) *N* (%)	3/10 (30.0)	15/15 (100.0)	5 (25.0)	48 (44.4)
Chosen the education at first hand (yes) *N* (%)	7/10 (70.0)	N/A	17 (85.0)	103 (95.3)
Would have applied if the program was not web-based (yes) *N* (%)	2/10 (20.0)	3 (20.0)	6 (30.0)	61 (56.5)
Combines studies with work/employment (yes) *N* (%)	9/10 (90.0)	11 (73.3)	11 (55.0)	N/A
Willing to move after graduation to work (yes) *N* (%)	7/10 (70.0)	7/14 (50.0)	13 (65.0)	N/A
In which area would you like to work after graduation? *N* (%)**				
Community pharmacy	3 (25.0)	3 (28.3)	16 (80.0)	88 (81.5)
Hospital pharmacy	4 (33.3)	5 (33.3)	5 (25.0)	8 (7.4)
Pharmaceutical industry	2 (16.7)	7 (46.7)	4 (20.0)	6 (5.6)
Government e.g., the medical agency	3 (25.0)	5 (33.3)	2 (10.0)	N/A
Hospital	1 (8.3)	4 (26.7)	1 (5.0)	N/A
PhD studies	3 (25.0)	2 (13.3)	3 (15.0)	2 (1.9)
Other ***	1 (8.3)	3 (28.3)	-	-

* Only mean value available [15]. ** Students chose several alternatives (although the question explicitly asked for one alternative), the total therefore exceeds 100%. *** Other includes clinical pharmacy and owning your own pharmacy, the alternative hospital could also include clinical pharmacy. N/A Not applicable because data was not available.

**Table 3:** Factors of importance when choosing the pharmacy education (mean value ± standard deviation). Students admitted to the Master of Science in Pharmacy program, Bachelor of Science in Pharmacy program and Master of Pharmaceutical Science program at Umeå University in August 2017. For comparison, data from students admitted to the Bachelor of Science in Pharmacy program in 2003 are shown. The students ranked the importance of the factor according to a 6-graded Likert scale as described in the methods section.

Factor	Master of Science in Pharmacy (*N* = 12)	Master of Pharmaceutical Science (*N* = 15)	Bachelor of Science in Pharmacy (*N* = 20)	Bachelor of Science in Pharmacy (*N* = 108) *
Interesting education	4.7 ± 0.5	4.6 ± 0.5	4.4 ± 0.7	4.3
Wide-ranging education	3.4 ± 1.6	4.3 ± 0.9	3.2 ± 1.2	N/A
Gives me personal development	3.8 ± 0.9	4.3 ± 0.7	4.3 ± 0.7	3.9
Leads to an interesting job	4.4 ± 0.8	4.5 ± 0.6	4.4 ± 0.7	4.5
The program is web-based	4.3 ± 1.1	3.7 ± 2.0	3.9 ± 0.7	2.4
Gives me a university degree	3.9 ± 1.6	3.8 ± 1.5	4.3 ± 0.9	2.2
Possibility to obtain a job near my hometown	3.8 ± 1.5	1.8 ± 2.0	4.1 ± 1.3	4.2
Salary after graduation	3.5 ± 1.2	3.0 ± 1.4	3.6 ± 0.8	N/A
Got recommendations about the program	3.1 ± 2.1	1.7 ± 1.9	2.3 ± 1.5	N/A
The university that is closest to my hometown	0.4 ± 0.9	0.4 ± 0.8	1.4 ± 1.9	N/A
Easy to travel to Umeå	1.4 ± 1.3	1.4 ± 1.3	2.5 ± 1.7	N/A

* Only mean values available [15]. N/A Not applicable because the questions were not included in the survey in 2003.
